# AN ASSESSMENT OF GERIATRIC TTRAINING AND SUPPORT NEEDS FOR CAREGIVERS AND HEALTH CARE PROFESSIONALS IN RURAL TEXAS

**DOI:** 10.1093/geroni/igz038.3390

**Published:** 2019-11-08

**Authors:** Susanna Luk-Jones, Jennifer J Severance, Doni Green, Donald Smith, Roslin Jose

**Affiliations:** 1 University of North Texas Health Science Center, Fort Worth, Texas, United States; 2 North Central Texas - Area Agency on Aging, Arlington, Texas, United States; 3 United Way's Area Agency on Aging of Tarrant County, Fort Worth, Texas, United States

## Abstract

Of the 254 counties in Texas, 69% are rural, and three out of every four counties are designated as whole or partial Primary Care Health Professional Shortage Areas. Rural counties in Texas have a higher proportion of older adults compared to metropolitan counties, and rural older adults with Alzheimer’s Disease and their caregivers face unique challenges of limited access to healthcare and lower earnings, resulting in more health-related problems. As part of a HRSA Geriatrics Workforce Enhancement Program, an academic medical center, two Area Agencies on Aging in North Texas, and an Alzheimer’s Association Chapter partnered to expand access to evidence-based programs into surrounding rural counties for older adults and caregivers of persons with Alzheimer’s Disease. An interdisciplinary workgroup developed focus group questionnaires for older adults, caregivers, and health care providers in rural areas to identify perceived needs, barriers to accessing services, and strategic partnerships. The North Central Texas Council of Governments conducted 11 focus groups in late 2018 and early 2019. Of these, seven consisted of family members caring for persons with memory loss. Four consisted of professionals who treat persons with memory loss. Transcription and thematic analysis identified key themes of training needs (both providers’ and laypersons’), resource needs, providers’ best practices, barriers to quality care, and other support needs. Practice implications of the findings include cross-sector partners and integrating telehealth platforms for program delivery. Collaboration between academic and community partners can expand access to evidence-based programs for rural and other underserved communities and address areas of need.

